# Assessment of the Microcirculation During Extracorporeal Blood Purification in Septic Patients: A Narrative Review

**DOI:** 10.3390/medicina62050879

**Published:** 2026-05-04

**Authors:** Darja Smirnova, Mara Klibus, Olegs Sabelnikovs

**Affiliations:** 1 Department of Anesthesiology, Intensive Care and Clinical Simulations, Riga Stradins University, LV-1007 Riga, Latvia; 2 Department of Anesthesiology and Reanimatology, Pauls Stradins Clinical University Hospital, LV-1002 Riga, Latvia

**Keywords:** direct microcirculation assessment, extracorporeal blood purification, sepsis, tissue perfusion, videomicroscopy

## Abstract

*Background and Objectives*: Microcirculatory dysfunction is a key feature of septic shock and contributes to organ failure despite the apparent normalization of systemic hemodynamic parameters. Extracorporeal blood purification (EBP) therapies aim to modulate the dysregulated inflammatory response through removal of endotoxins and cytokines. However, their impact on tissue-level perfusion remains unclear. Direct bedside assessment of microcirculation may provide mechanistic insight into the effects of EBP beyond macrohemodynamic stabilization. To date, no structured review has specifically synthesized evidence on direct microcirculatory assessment during EBP therapy in sepsis. *Materials and Methods:* This structured narrative review summarizes current evidence on direct microcirculatory assessment during EBP therapy in sepsis. A literature search of PubMed, Web of Science, and Scopus was performed using combinations of the terms “microcirculation” and “blood purification” or “hemoadsorption.” Studies published between 2015 and 2026 evaluating direct sublingual microcirculation using sidestream dark field (SDF) or incident dark field (IDF) videomicroscopy during EBP were included. Both experimental and clinical studies were considered. *Results*: Eight studies met the inclusion criteria. Selective endotoxin adsorption with polymyxin B hemoperfusion (PMX-HP) demonstrated improvements in perfused vessel density and small vessel density in both animal and clinical settings. Non-selective cytokine adsorption devices (CytoSorb and HA380) were associated with increases in microvascular flow index (MFI), perfused vessel density (PVD), and proportion of perfused vessels (PPV), although most data derive from small observational studies. Across studies, improvements in microcirculatory parameters were observed during or following hemoadsorption therapy. However, heterogeneity in design, timing, and concomitant treatments limits definitive interpretation. *Conclusions*: The included studies report improvements in microcirculatory parameters in septic patients during hemoadsorption therapy. However, despite signals suggesting improved perfusion, interpretation is limited by methodological constraints and the use of hemoadsorption within multimodal sepsis treatment. In the absence of adequately powered randomized controlled trials, these changes cannot be directly attributed to mediator removal and should be considered within the broader context of overall therapy.

## 1. Introduction

Septic shock is a life-threatening condition characterized by a dysregulated immune response to infection, resulting in profound circulatory, cellular, and metabolic disturbances. These alterations lead to both macro- and microhemodynamic dysfunction, impaired tissue perfusion, and ultimately multi-organ failure [1,2,3,4], making microcirculatory dysfunction a key pathophysiological feature of septic shock. Moreover, persistent microvascular alterations may contribute to ongoing organ dysfunction even when global hemodynamics appear stabilized [5,6,7,8,9].

Extracorporeal blood purification (EBP) therapies have been introduced as adjunctive treatments in septic shock with the aim of modulating the dysregulated inflammatory response [10,11,12]. Although conclusive evidence demonstrating improved clinical outcomes remains limited, these therapies continue to be widely adopted in clinical practice [13,14]. Several studies have reported reductions in vasopressor requirements and improvements in selected macrohemodynamic parameters [15,16,17,18]. However, whether such systemic changes translate into recovery of the microcirculation remains unclear [12,19].

Microcirculatory assessment during EBP has most commonly relied on indirect markers, such as lactate clearance. However, lactate is not a direct measure of microvascular perfusion. Therefore, its reliability as an indicator of true microcirculatory recovery is limited. In contrast, direct visualization techniques, such as sidestream dark field (SDF) and incident dark field (IDF) videomicroscopy, allow quantitative evaluation of capillary flow and have demonstrated clinical utility [20,21,22,23]. By removing circulating cytokines, endotoxins, and other inflammatory mediators, EBP devices are theoretically capable of influencing microvascular function [24]. For these therapies to confer meaningful clinical benefit, their effects should extend beyond systemic stabilization to the restoration of effective tissue perfusion. Given the association between microcirculatory impairment and adverse outcomes in sepsis, evaluating microvascular responses during EBP therapy may provide important mechanistic and prognostic insights [8,12]. Monitoring changes in the microcirculation could help guide the initiation, duration, and termination of EBP therapy—particularly in settings where advanced biochemical immunomonitoring is not readily available [12,19]. Despite this rationale, there is currently no standardized framework for incorporating microcirculation monitoring into clinical decision-making during EBP therapy [25,26,27]. 

Given the central role of microvascular dysfunction in septic organ failure, a clearer understanding of how EBP influences tissue-level perfusion is needed. This narrative review aims to critically evaluate clinical studies investigating microcirculatory changes during extracorporeal blood purification in patients with sepsis. By synthesizing the available evidence and identifying methodological limitations and knowledge gaps, we aim to clarify the current state of evidence and its implications for clinical practice. This manuscript also outlines the impact of inflammatory mediators, particularly endotoxins and cytokines, on the microcirculation. In addition, it summarizes available EBP techniques and bedside monitoring methods. Together, these provide the necessary background for interpreting clinical findings in patients with septic shock.

## 2. Materials and Methods

### Search Strategy and Study Selection

This narrative review was prepared in accordance with SANRA principles for narrative reviews [28]. A structured literature search was conducted in three electronic databases: PubMed, Web of Science, and Scopus. The search included studies published between January 2015 and March 2026. The search strategy combined controlled vocabulary (Medical Subject Headings, MeSH, in PubMed) and free-text terms related to microcirculation, direct assessment tools, sepsis, and extracorporeal blood purification. To ensure transparency and reproducibility, the full database-specific search strategies are provided in the Supplementary Materials (Table S1).

The search aimed to identify studies evaluating microcirculatory alterations in septic patients undergoing EBP therapy. Particular focus was placed on studies using direct assessment of the microcirculation, such as SDF or IDF videomicroscopy. Broader search terms were retained to minimize the risk of missing relevant studies due to variability in terminology and indexing.

Titles and abstracts were screened for relevance, followed by full-text assessment of potentially eligible articles. The screening process was performed independently by two reviewers, with disagreements resolved through discussion and consensus.

Studies were included if they met the following criteria: (1) publication in English; (2) availability of full text; (3) publication between 2015 and 2026; and (4) evaluation of direct microcirculation in the context of EBP therapy in septic patients. Eligible study designs included observational, interventional, experimental studies, and relevant case reports.

To ensure comprehensive study identification, the database search was supplemented by manual screening of reference lists of included articles and relevant review papers, as well as targeted searches using specific device names (e.g., CytoSorb, HA380, polymyxin B hemoperfusion) and related articles suggested by database algorithms.

The final selection of articles was based on relevance to the objectives of the review, as determined through consensus among the reviewers. This review does not aim to provide an exhaustive systematic synthesis but rather to critically summarize the available evidence and highlight key methodological considerations, principal findings, and existing knowledge gaps.

## 3. Pathophysiological and Methodological Background

### 3.1. Microcirculatory Change in Septic Shock

Septic shock results from complex interactions between the pathogen and a dysregulated host immune response. This leads to excessive inflammation followed by immunosuppression and immune paralysis [3,4]. A dysregulated inflammatory response disrupts the coordinated function of large (macro) and small (micro) blood vessels, a phenomenon referred to as loss of hemodynamic coherence [5,6,7]. Sepsis-induced microcirculatory disturbances are characterized by reduced capillary density and increased perfusion heterogeneity, resulting from dysregulated vasodilation and vasoconstriction [6,7,8,9,10]. These alterations impair oxygen delivery to tissues and contribute to organ dysfunction and disease progression [5,8].

The resuscitation strategy recommended in international guidelines [29] for septic shock has historically focused on improving macrocirculation. Although the ultimate goal is to normalize microcirculation and maintain end-organ perfusion, the primary targets have been restoration of mean arterial pressure (MAP) and cardiac output. While a pre-defined MAP above 65 mmHg is the most common approach, recent evidence suggests that MAP may not correlate with adequate blood flow through the end capillaries (microcirculation) and that microcirculatory dysfunction may exist despite a normal MAP. This means that even if the blood pressure (macrocirculation) normalizes, the microcirculatory dysfunction persists and leads to organ failure [5]. For this reason, microcirculatory assessment tools have been developed, although microcirculatory function is not currently routinely monitored at the bedside.

De Backer et al. (2013) [30] demonstrated that microvascular and macrovascular parameters may operate independently. They also showed that microcirculatory alterations are more pronounced in the early phase of sepsis and that the proportion of perfused small vessels—reflecting perfusion heterogeneity—is an independent predictor of outcome. Similar results were reported in an animal study by Zhang et al. which showed that microcirculation and macrocirculation behave independently in endotoxemic shock, with disturbances in microcirculation occurring before changes in macrocirculation parameters [31]. In a study by Fage et al., fluid-responsive patients demonstrated a significant increase in cardiac output following fluid administration and an increase in MAP after noradrenaline infusion. However, the capillary refill time (CRT) response was variable, decreasing in some patients while remaining unchanged in others. This finding highlights a dissociation between macro- and microhemodynamic variables [32]. In contrast, other studies have reported a positive correlation between macro- and microcirculatory parameters in septic shock, particularly during the early phase of treatment [33,34].

#### The Role of Inflammatory Mediators in Microcirculatory Dysfunction

The exaggerated host response in sepsis can lead to both structural and functional damage to the endothelium—the key regulator of microvascular homeostasis. Critical endothelial functions become impaired. This contribute to the formation of microthrombi, tissue edema, interstitial fluid leakage and dysregulation of vascular tone [8,9,10]. As a result, the primary function of the microcirculation—delivering oxygen from red blood cells to tissue cells—becomes impaired [25,33].

In gram negative sepsis, the excessive release of cytokines is triggered by lipopolysaccharide (LPS, endotoxin), an integral component of the outer membrane of bacteria [35]. Endotoxin per se can induce a range of microcirculatory alterations by promoting a proinflammatory endothelial phenotype. This includes glycocalyx damage with subsequent loss of endothelial integrity, dysregulation of vascular tone due to excessive nitric oxide (NO) production, and coagulopathy [36]. Endotoxin may also be present in gram-positive septic shock, potentially due to bacterial translocation from an ischemic gut. Even at relatively low doses, endotoxin can significantly impair the immune system [37], while higher levels are strongly associated with the development of septic shock [38,39]. The subsequent overproduction of pro-inflammatory cytokines further exacerbates endothelial damage and leads to microcirculatory disturbances [6,8].

The enhanced immune response contributes to a toxic concentration that leads to multiorgan failure, coagulopathy and hyperlactatemia [40]. This supports the concept that inflammatory mediators, such as endotoxin and cytokines, play a key role in disrupting hemodynamic coherence in septic shock. Therapeutic approaches aimed at reducing mediator levels below toxic thresholds may help restore the interplay between micro- and macrohemodynamics [20].

### 3.2. Extracorporeal Blood Purification Techniques in Septic Shock

Various extracorporeal blood purification therapies have been proposed as supportive treatments in septic shock, aiming to reduce excessive inflammation and restore physiological homeostasis [11,41]. In this context, EBP is primarily based on the physical principle of adsorption rather than diffusion or convection. It is therefore often referred to as hemoadsorption (previously termed hemoperfusion). Hemoadsorption (HA) therapies can be classified as targeted (selective) or broad-spectrum (non-selective) based on their solute removal properties. They may be performed as stand-alone treatments or in combination with continuous renal replacement therapy (CRRT). Broad-spectrum technologies act on multiple solutes simultaneously, whereas targeted technologies aim to remove specific molecules, such as endotoxin [13,42].

The most commonly used non-selective EBP devices include the Oxiris^®^ membrane (Vantive, IL, USA), which adsorbs both cytokines and endotoxins. Other widely used devices are the CytoSorb^®^ cartridge (CytoSorbents Corporation, Princeton, NJ, USA) and the HA380 cartridge (Jafron Biomedical, China), both of which provide non-selective cytokine adsorption. The only widely used selective EBP therapy currently available is polymyxin B hemoperfusion (PMX-HP; Toray Industries Inc., Tokyo, Japan), which specifically targets endotoxin removal [11,13]. In recent years, additional adsorption devices have been introduced, including the Efferon^®^ LSP cartridge (Efferon OÜ, Tallinn, Estonia), designed for selective endotoxin removal [18], and the CA330 series cartridges (Jafron Biomedical Co., Zhuhai, China), developed for cytokine adsorption in septic patients [19]. However, clinical data supporting their widespread use and effectiveness in routine practice remain limited.

Anticoagulation is generally required for the delivery of EBP therapy, unless contraindicated. In clinical practice, this is most commonly achieved either by systemic anticoagulation with heparin or by regional anticoagulation with citrate [13]. PMX-HP requires systemic anticoagulation, whereas HA combined with CRRT may be performed using either citrate or systemic heparin.

Despite its promising potential, the efficacy of EBP therapies in septic shock patients remains controversial, as large randomized controlled trials have not demonstrated a clear long-term survival benefit [14,17]. Major challenges include suboptimal timing for the initiation and termination of hemoadsorption, premature saturation of hemoadsorption devices, and inadequate patient selection due to the lack of effective immunomonitoring methods [11,13,14]. Although the removal of target solutes—such as endotoxins or cytokines—is the primary goal of initiating EBP therapy, the measurement of these molecules is often limited by the availability, accuracy and cost of the tests [38,40]. Therefore, treatment decisions are typically based on clinical indicators such as hemodynamic instability, organ dysfunction and microcirculatory impairment. However, there is currently no standardized approach for integrating advanced microcirculation monitoring techniques into clinical decision-making during EBP therapies, and clinicians continue to rely on visual or biochemical markers of microcirculatory dysfunction [13,43].

### 3.3. Microcirculatory Assessment in Septic Shock and Its Relevance During Blood Purification

Given the loss of coherence between micro- and macrohemodynamics in sepsis, management strategies in septic shock should incorporate assessment of the microcirculation rather than relying solely on systemic hemodynamic variables [6,8]. Historically, the most commonly used approaches for evaluating microcirculation in clinical practice have been visual bedside assessment and biochemical markers [1]. However, both provide only indirect information regarding microvascular function.

Visual assessment methods—including the skin mottling score (SMS) and capillary refill time (CRT)—offer simple and non-invasive estimates of peripheral perfusion and have demonstrated prognostic relevance in septic shock [44,45,46]. Nevertheless, these tools remain semi-quantitative and operator-dependent. The ANDROMEDA-SHOCK-2 trial [47] described a standardized approach to CRT assessment, aiming to improve measurement consistency and reduce variability. Furthermore, automated CRT systems have been developed to minimize observer bias by applying controlled pressure and using optical sensors to detect reperfusion following blanching [48,49]. Although these technologies may improve reproducibility, current evidence is limited by small sample sizes, and large-scale clinical validation is still lacking.

The biochemical approach primarily relies on serial serum lactate measurements and lactate clearance. Lactate remains one of the most widely used biomarkers of tissue hypoperfusion in septic shock [1,50]. However, lactate reflects global metabolic stress rather than direct microvascular flow and lacks specificity for microcirculatory dysfunction. Moreover, in the context of extracorporeal blood purification—particularly when HA is combined with renal replacement modalities—lactate levels may be influenced by extracorporeal removal, potentially limiting their reliability as indicators of true microcirculatory recovery.

Given the limitations of visual and biochemical approaches [51,52,53,54], optical imaging techniques have increasingly been adopted in research practice over recent decades [25,27]. These include near-infrared spectroscopy (NIRS), photoplethysmography-based indices, and videomicroscopy. NIRS measures regional tissue oxygen saturation, reflecting the balance between oxygen delivery and consumption at the tissue level. Photoplethysmography-derived indices, such as the perfusion index or pulse amplitude index, estimate peripheral perfusion by quantifying pulsatile blood flow within the microvascular bed. While NIRS and photoplethysmography provide indirect information on tissue oxygenation or peripheral perfusion, they do not directly visualize capillary blood flow. Thus, videomicroscopy currently represents the only direct bedside method for assessing microcirculation in clinical research.

Direct microcirculatory assessment relies on high-resolution videomicroscopy techniques, such as sidestream dark field (SDF) and incident dark field (IDF) imaging [25]. These methods allow in vivo visualization of the microvascular network and quantitative measurement of capillary density, perfusion, and flow dynamics. Key parameters include small vessel density (SVD), total vessel density (TVD), perfused vessel density (PVD), proportion of perfused vessels (PPV), microvascular flow index (MFI), and flow heterogeneity index (HI). SVD and TVD reflect the structural density of the capillary network, whereas PVD and PPV represent the functional component of perfusion by quantifying the proportion and density of vessels with continuous blood flow. MFI provides a semi-quantitative assessment of flow quality using a score from 0 (no flow) to 3 (normal continuous flow), while the HI describes the spatial variability of perfusion between different quadrants of the imaging field. Impaired microcirculation is characterized by reduced functional capillary density, decreased perfused vessel proportion, heterogeneous or intermittent flow, and increased flow heterogeneity—findings that are associated with organ dysfunction and adverse outcomes in septic shock [29]. In clinical research, microcirculatory assessment using videomicroscopy has been most extensively performed at the sublingual site, where the thin epithelial layer permits optimal image acquisition and consistent visualization of capillary flow [7,55]. A recent meta-analysis (2024) found that impaired microcirculatory parameters assessed by videomicroscopy are independently associated with increased mortality and greater illness severity in sepsis, supporting its clinical and prognostic relevance [56]. However, it remains uncertain to what extent sublingual findings can be directly extrapolated to other internal organs. While some studies suggest a correlation with systemic perfusion disturbances [50,57], these associations should be interpreted as indicative rather than definitive, reflecting a signal rather than a direct representation of whole-body microcirculatory status.

Given these methodological differences and the limitations of surrogate markers, the present review places particular emphasis on studies employing direct optical assessment of the microcirculation when evaluating the impact of extracorporeal blood purification therapies.

## 4. Key Findings

In accordance with the predefined search strategy (Table S1), 46 records were identified through PubMed, 42 through Web of Science, and 84 through Scopus. After removal of duplicate records, the remaining articles underwent a two-step screening process. Titles and abstracts were first assessed for relevance, followed by full-text evaluation to determine methodological eligibility.

To be included in the analysis, studies were required to evaluate direct microcirculatory assessment using SDF or IDF videomicroscopy in a sepsis population or related syndromes (e.g., multisystem inflammatory syndrome), including adult, pediatric, and animal models. In addition, patients had to be treated with hemoadsorption devices during microcirculatory assessment period, either as stand-alone therapies or in combination with CRRT. Studies were excluded if they used indirect methods of microcirculatory assessment (e.g., lactate levels, Doppler ultrasonography), involved non-sepsis conditions (e.g., cardiogenic shock, rhabdomyolysis), applied other extracorporeal support modalities without hemoadsorption (e.g., conventional high-flux hemodialysis), or if relevant information was not reported in the abstract. After applying these criteria, a total of eight studies met the inclusion requirements and were selected for detailed analysis.

The characteristics and principal findings of the eight included studies are summarized in Table 1. The table presents the study design, type of EBP therapy, method of direct microcirculatory assessment, evaluated microcirculatory parameters, and the main finding and limitations.

Below is a list of variables used to assess microcirculation with brief explanations, mentioned in the referenced studies.

- **Total Vessel Density (TVD, mm/mm^2^):** The TVD is calculated as the total length of vessels divided by the total surface area of the region of interest. It reflects the structural vessel density within the observed microvascular network, independent of flow status.
- **Small Vessel Density (SVD, mm/mm^2^):** SVD represents the total length of small vessels (typically ≤20 μm in diameter) divided by the total surface area of the region of interest. It specifically quantifies capillary-level vessel density and is particularly relevant in septic shock, where small-vessel alterations are most pronounced.
- **Perfused Vessel Density (PVD, mm/mm^2^):** an indicator of functional vessel density and is determined as the total length of perfused vessels divided by the total surface area. It reflects the density of vessels with continuous blood flow and is often used as a surrogate for functional capillary density.
- **Proportion of Perfused Vessels (PPV, %):** reflects the overall quality of microvascular perfusion and is calculated as the number of perfused vessels divided by the total number of vessels, expressed as a percentage. It represents the proportion of vessels that are effectively perfused within the observed field.
- **Microvascular Flow Index (MFI):** a semi-quantitative measure of flow quality in microcirculation. The imaging field is divided into four quadrants, each scored from 0 to 3 based on observed flow characteristics (0 = no flow, 1 = intermittent flow, 2 = slow flow, 3 = continuous flow). The final MFI is calculated as the average of these scores, providing an overall assessment of microvascular perfusion.

As mentioned above, videomicroscopy provides a direct bedside method for assessing real-time microcirculatory changes in critically ill patients, including those undergoing EBP. In this review, all included studies used SDF or IDF videomicroscopy and reported changes in microcirculatory parameters during hemoadsorption therapy. The evidence base comprised two randomized controlled trials, two prospective observational studies, one retrospective observational study, two case reports, and one animal study.

In a recent randomized controlled trial, Chen et al. (2020) [60] reported improvements in microcirculatory parameters in patients with septic shock treated with selective endotoxin hemoadsorbent PMX-HP. The main finding of this study was that total vessel density (TVD) and perfused vessel density (PVD) were higher in the PMX-HP group than in the control group. These findings are consistent with earlier investigations by Yeh et al. In an experimental septic pig model, Yeh et al. (2017) [59] observed higher perfused small vessel density and tissue oxygen saturation within 6 h in the PMX-HP group compared with conventionally treated sepsis, and noted an association with reduced histologic organ injury. Similarly, in a multicenter RCT study of patients with severe sepsis and septic shock, Yeh et al. (2015) [58] reported significantly higher small vessel density and perfused small vessel density at 48 h in patients treated with PMX-HP compared with controls.

In a prospective observational study, Zuccari et al. (2020) [61] assessed microcirculation using SDF videomicroscopy and NIRS in septic patients undergoing non-selective cytokine hemoadsorption with the CytoSorb cartridge during continuous renal replacement therapy. Changes in microcirculation at the sublingual site were assessed at baseline, 6 h, and 24 h after the start of the procedure. The study observed changes in microcirculatory parameters, particularly in PVD at 6 and 24 h and in TVD at 24 h. In contrast, no changes were detected in NIRS-derived parameters.

Evidence describing microcirculatory changes during CytoSorb therapy has also been reported in several individual clinical cases. Duran et al. (2022) [63] used IDF videomicroscopy in an adult patient with abdominal sepsis undergoing hemoadsorption therapy to assess microcirculation. The reported data showed severe baseline microcirculatory impairment, with subsequent progressive changes in MFI and PVD during the observation period. Similarly, Bottari et al. (2021) [62] described a pediatric case of severe multisystem inflammatory syndrome. Serial IDF imaging revealed marked microcirculatory alterations during the first 96 h, followed by delayed improvement of key parameters despite early macrocirculatory stabilization. This observation suggested a lack of hemodynamic coherence in the early phase. The improvement in microcirculation parameters during CytoSorb treatment was further reported in a larger pediatric cohort by Bottari et al. (2024) [65]. In this single-center pilot study of critically ill children with septic shock receiving adjunctive hemoadsorption therapy, sublingual microcirculation was assessed using IDF videomicroscopy. The study showed statistically significant improvements in microcirculatory parameters, particularly an increase in microvascular flow index and proportion of perfused vessels.

Improvements in microcirculatory parameters such as MFI and PPV were described in a retrospective observational study by Zhu et al. (2024) [64]. The study included adult patients with sepsis who received the non-selective cytokine adsorber HA380 as adjunctive therapy in combination with CRRT. Microcirculatory parameters were assessed at baseline and after seven days. The retrospective analysis showed that, after 7 days of treatment, both MFI and PPV were significantly higher in the intervention group compared with controls.

## 5. Discussion

Microcirculatory dysfunction is a central pathophysiological hallmark of septic shock. It is closely linked to endothelial injury, excess inflammatory mediators, coagulation disturbances, and impaired oxygen extraction [6,7,8,9,10,36]. This results in persistent alterations in functional capillary density, perfusion heterogeneity, and flow quality, which contribute to organ failure and are associated with adverse clinical outcomes in sepsis.

Extracorporeal blood purification therapies have been introduced with the aim of attenuating the dysregulated inflammatory response by removing endotoxins and/or circulating cytokines [11,12,13]. Although several trials and meta-analyses report improvements in macrohemodynamic stability [16,17,66], their translation into microvascular recovery remains uncertain. Lactate reduction, although frequently interpreted as a surrogate of improved tissue perfusion [51], may be influenced by extracorporeal clearance mechanisms, particularly when hemoadsorption is combined with renal replacement therapy [11,12]. Therefore, reliance on lactate alone may overestimate the true recovery of microcirculatory function.

The present review specifically focused on studies that directly assessed microcirculation during EBP therapy using videomicroscopy techniques. All included studies evaluated sublingual microcirculation using sidestream dark field (SDF) or incident dark field (IDF) imaging, currently the only bedside techniques allowing direct real-time visualization of capillary flow [25,26]. Across selective endotoxin adsorption (PMX-HP) and non-selective cytokine adsorption devices (CytoSorb and HA380), changes in functional microcirculatory parameters—particularly PVD, PPV, and MFI—have been reported across studies [58,59,60,61,62,63,64,65].

In studies evaluating selective endotoxin removal with PMX-HP, changes in microcirculatory parameters were reported both experimentally and in randomized controlled trials. Animal data showed early increases in perfused small vessel density and tissue oxygenation [59], while clinical studies reported higher small vessel density and perfused vessel density compared with the control group [58,60]. PMX-HP is the only hemoadsorption device evaluated as a stand-alone modality in randomized controlled trials within the studies included in this review. The findings of these studies are consistent with the role of endotoxin-driven endothelial dysfunction in microvascular impairment [36,37,38].

Similar findings were observed in observational studies, case reports, and interventional trials assessing microcirculation in septic patients undergoing non-selective cytokine adsorption therapies. In septic patients treated with CytoSorb, increases in PVD and TVD were reported [61], as shown in a small single-center observational study. Both individual case reports included in this review described delayed changes in microcirculatory parameters despite early macrohemodynamic stabilization [62,63]. This observation is consistent with the phenomenon of hemodynamic incoherence; however, it should be interpreted with caution given the methodological limitations of case reports.

The effect of the HA380 cartridge on microcirculatory parameters measured by SDF was evaluated in a retrospective study including 54 patients with sepsis treated with HA380 in combination with CRRT [64]. In that study, MFI and PPV were higher after seven days of treatment in the HA380 group compared with the control group.

Despite the findings described above, the results should be interpreted with caution. Videomicroscopy is a promising approach in the management of critically ill patients and provides high-resolution, physiologically relevant information. However, improvements in microcirculatory parameters assessed at the sublingual site cannot be directly translated into restoration of microcirculation across different organ beds. Furthermore, there is currently no convincing evidence that such changes in microcirculatory parameters correspond to meaningful clinical benefit or improved patient-centered outcomes. In addition, videomicroscopy has certain technical and methodological limitations, including operator dependency, variability in image acquisition and analysis, and potential pressure- or saliva-related artifacts, which should be considered when interpreting the findings.

Despite the overall consistency of reported changes in microcirculatory parameters assessed by videomicroscopy, several limitations should be considered in the studies included in this analysis. First, no formal risk-of-bias assessment tool was applied; however, methodological limitations of the included studies were considered during data interpretation. This should be taken into account when interpreting the findings, as it may limit the ability to systematically evaluate study quality and the overall strength of the evidence. Second, most studies were small, single-center, or observational in design, resulting in reduced statistical power and generalizability. In addition, the limited availability of randomized controlled trials and heterogeneous study populations further weaken the strength of the evidence and increase the risk of selection bias. Third, an important methodological distinction across the included studies relates to the mode of extracorporeal therapy. While endotoxin adsorption studies (e.g., polymyxin B hemoperfusion) were predominantly performed as stand-alone interventions, most studies investigating cytokine adsorption devices (e.g., CytoSorb, HA380) applied hemoadsorption in combination with continuous renal replacement therapy. This heterogeneity limits direct comparability and complicates attribution of microcirculatory effects to adsorption alone. In most studies, the specific CRRT modality (e.g., CVVH versus CVVHDF) was not clearly specified. Given that cytokines are predominantly middle-molecular-weight solutes and are more effectively removed by convective clearance, as in CVVH, variability in CRRT modality may significantly influence the overall efficacy of extracorporeal blood purification and confound interpretation of the observed effects. Furthermore, anticoagulation strategies were not consistently reported, limiting the assessment of their potential impact on microcirculatory outcomes. In particular, the contribution of systemic heparin to microthrombosis prevention cannot be adequately evaluated. The absence of standardized protocols for timing, duration, and monitoring of EBP therapy further reduces comparability across studies and limits external validity. Fourth, hemoadsorption was frequently administered alongside multimodal sepsis therapy, including fluid resuscitation and antimicrobial treatment. Consequently, improvements in microcirculation cannot be attributed solely to mediator removal and should be interpreted with caution.

## 6. Future Perspectives

Future studies should determine whether improvements in sublingual microcirculatory parameters assessed by videomicroscopy correlate with clinically relevant outcomes, such as microcirculatory improvement in other organ beds and survival. This would help bridge the gap between physiological measurements and patient-centered outcomes. At the same time, extracorporeal therapy–related variables, including CRRT modality and anticoagulation strategy, should be systematically evaluated, as they may independently influence microvascular perfusion.

Therefore, future research should incorporate predefined microcirculatory endpoints into adequately powered randomized controlled trials to determine whether EBP-induced changes translate into meaningful clinical benefit. Standardization of image acquisition and analysis is essential to ensure reproducibility and facilitate clinical implementation. If validated, direct microcirculatory monitoring may enable precision-based approaches, including patient selection, optimization of treatment timing and duration, and timely replacement of adsorption devices.

## 7. Conclusions

In summary, current evidence suggests that extracorporeal blood purification may improve microvascular parameters assessed by direct videomicroscopy in patients with septic shock. However, the available data remain limited, heterogeneous, and insufficient to establish a causal relationship between mediator removal through hemoadsorption and sustained microcirculatory recovery. Although direct microcirculatory monitoring offers valuable physiological insights and potential guidance for EBP therapy, its routine clinical implementation requires validation in standardized, adequately powered randomized controlled trials.

## Figures and Tables

**Table 1:** Review of clinical studies in chronological order using optical imaging techniques for direct microcirculation assessment during blood purification therapies.

Study	Study Design, Sample Size	Hemoadsorption Therapy	Microcirculation Assessment tool	Timing of Assessment	Main Findings	Key Methodological Limitation
**Yeh et al. (2015)** [58]	Multicenter Randomized Controlled Trial (RCT); 13 adult patients; 7 PMX-HP and 6 control group	Selective endotoxin adsorption with Polymyxin B-immobilized fiber column (**PMX-HP**); stand-alone mode; systemic heparinization	SDF videomicroscopy	Baseline (0 h), 24 h and 48 h	Total SVD higher at 48 h and perfused SVD higher at 24 h and 48 h in PMX-HP vs. control (*p* = 0.001; *p* = 0.007 and *p* < 0.001, repsectively).	Small sample size; limited patient data reporting
**Yeh et al. (2017)** [59]	Experimental animal (n = 18 pigs; 3 groups: sham, sepsis, sepsis + PMX-HP; n = 6 per group)	**PMX-HP,** stand-alone mode; systemic heparinization	SDF videomicroscopy and tissue oxygen saturation	0 h and 6 h	Higher perfused SVD and tissue oxygen saturation at 6 h in PMX-HP+sepsis vs. sepsis group (*p* < 0.05)	Short observation period (6 h); animal model limits clinical translation
**Chen et al. (2020)** [60]	RCT (28 adults included; 14 in interventional group and 14 in control group)	**PMX-HP,** stand-alone mode; systemic heparinization	SDF videomicroscopy	0 h, 24 h and 48 h	Higher TVD (*p* = 0.007) and PVD (*p* = 0.008) at 48 h in the PMX-HP group compared to the control group.	Small sample size; single-center study
**Zuccari et al. (2020)** [61]	Prospective Observational Study (9 adults included)	Unselective cytokines adsorption with **CytoSorb** and CRRT (modality and anticoagulation strategy not reported)	SDF videomicroscopy and NIRS with vascular occlusion test	0 h, 6 h and 24 h	Microvascular perfusion indices changed over time, with a significant increase in PVD at 6 and 24 h (*p* = 0.003) and in TVD at 24 h (*p* = 0.0015) compared with baseline. No significant changes were observed in NIRS-derived parameters related to tissue oxygenation or microvascular reactivity.	Very small cohort; observational design; CRRT confounding
**Bottari et al. (2021)** [62]	Case report (pediatric patient with severe MIS-C)	**CytoSorb** + CRRT (CVVHD, anticoagulation strategy not reported)	IDF videomicroscopy	Serial, 12–120 h (24 h intervals)	Serial IDF imaging showed marked microcirculatory impairment during the first 96 h (MFI < 2.75, reduced TVD, PPV, PVD). Despite early hemodynamic recovery, principal microcirculatory parameters improved only after 96 h (day 5), indicating delayed restoration of microvascular perfusion	Single case; multimodal therapy (immunomodulation therapy; confounding effect of CRRT); no control group; limited generalizability
**Duran et al. (2022)** [63]	Case report (adult with abdominal sepsis)	**CytoSorb** + CRRT (modality and anticoagulation strategy not reported)	IDF videomicroscopy	0 h, 24 h, 36 h, 48 h, 120 h	IDF imaging showed severe baseline microcirculatory impairment with subsequent improvement in MFI and PVD during hemoadsorption therapy	Single case; confounding effect of CRRT; limited generalizability
**Zhu et al. (2024)** [64]	Retrospective single-center study (107 adults included; 54 patients in interventional group and 53 patients in control group)	Unselective cytokines adsorption with **HA380** hemoperfusion cartridge + CRRT (CVVHDF, heterogeneous anticoagulation strategies across the study population)	SDF videomicroscopy	0 h and 7 day	In the HA380 group, MFI and PPV were significantly higher after 7 days of treatment compared with the control group (*p* < 0.01)	Retrospective design; Confounding effect of CRRT
**Bottari et al. (2024)** [65]	Single-Center Observational Study/pilot study (13 pediatric patients included)	**CytoSorb** + CRRT (CVVHDF, systemic heparinization)	IDF videomicroscopy	0 h, 24 h, 48 h, 72 h, 96 h	Changes in microcirculatory parameters were observed in 10 of 13 patients undergoing hemoadsorption therapy, with a significant increase in MFI (*p* = 0.01) and PPV (*p* = 0.04).	Small sample; single-center; no control group; Confounding effect of CRRT

Abbreviations: CRRT (continuous renal replacement therapy); CVVHDF (continuous veno-venous hemodiafiltration); CVVHD (continuous veno-venous hemodialysis); (IDF) (Incident Dark Field videomicroscopy), MIS-C (multisystem inflammatory syndrome); MFI (microvascular flow index); NIRS (Near-Infrared Spectroscopy), PMX-HP (Polymyxin B-immobilized fiber column), PVD (perfused vessel density), PPV (proportion of perfused vessels), SDF (Sidestream Dark Field videomicroscopy), SVD (small vessel density), TVD (total vessel density).

## Data Availability

The original contributions presented in this study are included in the article’s material; further inquiries can be directed to the corresponding author.

## Supplementary Materials

The following supporting information can be downloaded at: https://www.mdpi.com/article/10.3390/medicina62050879/s1, Table S1: Detailed database search strategy.

## Abbreviations

The following abbreviations are used in this manuscript:

CRT	Capillary Refill Time
CRRT	Continuous renal replacement therapy
CVVHDF	continuous veno-venous hemodiafiltration
CVVHDF	Continuous Veno-Venous Hemodiafiltration
EBP	Extracorporeal Blood Purification
HA	Hemoadsorption
HI	Heterogeneity Index
IDF	Incident Dark Field videomicroscopy
LPS	Lipopolysaccharide
MAP	Mean Arterial Pressure
MFI	Microvascular Flow Index
MIS-C	Multisystem Inflammatory Syndrome in Children
NIRS	Near-Infrared Spectroscopy
PAI	Pulse Amplitude Index
PI	Perfusion Index
PMX-HP	Polymyxin B-Immobilized Fiber Column Hemoperfusion
PPV	Proportion of Perfused Vessels
PVD	Perfused Vessel Density
SDF	Sidestream Dark Field videomicroscopy
SMS	Skin Mottling Score
SVD	Small Vessel Density
TVD	Total Vessel Density
