# Supplementary material for: Assessment of the Microcirculation During Extracorporeal Blood Purification in Septic Patients: A Narrative Review
Source: Medicina (Kaunas). 2026 May 4;62(5):879. doi: 10.3390/medicina62050879 (PMC13208964; doi:10.3390/medicina62050879)
Supplement: Supplementary file 1 [file medicina-62-00879-s001.zip › medicina-4267628-supplementary.pdf]

Table S1. Detailed database search strategy

The following search strategies were used to identify studies evaluating microcirculatory alterations in septic patients undergoing extracorporeal blood purification (EBP) therapy.

PubMed (MEDLINE)

Search conducted using Medical Subject Headings (MeSH) and free-text terms: (("Microcirculation"[Mesh] OR microcirculation OR microvascular OR "sublingual microcirculation") AND ("Sepsis"[Mesh] OR sepsis OR "septic shock") AND ("Hemoperfusion"[Mesh] OR "Renal Replacement Therapy"[Mesh] OR "blood purification" OR hemoadsorption OR hemadsorption OR hemoperfusion OR "renal replacement therapy" OR CRRT OR CytoSorb OR HA380 OR Oxiris OR "polymyxin B hemoperfusion" OR PMX))

Limits applied:

- Publication date: January 2015 – March 2026
- Language: English

Scopus

The search was performed using the TITLE-ABS-KEY field, which includes title, abstract, and keywords.

The following search strategy was applied: TITLE-ABS-KEY(microcirculation OR microvascular OR "sublingual microcirculation") AND TITLE-ABS-KEY(sepsis OR "septic shock") AND TITLE-ABS-KEY("blood purification" OR hemoadsorption OR hemadsorption OR hemoperfusion OR "renal replacement therapy" OR CRRT OR CytoSorb OR HA380 OR Oxiris OR "polymyxin B hemoperfusion" OR PMX)

Limits applied:

- Publication date: January 2015 – March 2026
- Language: English

Web of Science

The search was performed using the Topic (TS) field, which includes title, abstract, author keywords, and Keywords Plus. The following search strategy was applied: TS=(microcirculation OR microvascular OR "sublingual microcirculation") AND TS=(sepsis OR "septic shock") AND TS=("blood purification" OR hemoadsorption OR hemadsorption OR hemoperfusion OR "renal replacement therapy" OR CRRT OR CytoSorb OR HA380 OR Oxiris OR "polymyxin B hemoperfusion" OR PMX)

Limits applied:

- Publication date: January 2015 – March 2026
- Language: English

Notes: The search strategy combined controlled vocabulary and free-text terms to account for variability in terminology and indexing. Device-specific keywords (e.g., CytoSorb, HA380, polymyxin B hemoperfusion) were included to ensure comprehensive identification of relevant studies.
